# dFlpTag, a RMCE-based tool for simultaneous endogenous protein tagging and cell labeling in *Drosophila*

**DOI:** 10.1038/s41598-025-30193-0

**Published:** 2025-11-27

**Authors:** Ling Li, Jiekun Yan, Yina Ruan, Miranda C. Dyson, Fran Laenen, Sha Liu, Xiaojun Xie

**Affiliations:** 1 Children’s Hospital, Zhejiang University School of Medicine, National Clinical Research Center for Child Health, National Children’s Regional Medical Center, Hangzhou, 310058 Zhejiang China; 2 Center for Brain & Disease Research, VIB-KU Leuven, Leuven, 3000 Belgium; 3 Department of Neurosciences, KU Leuven, Leuven, 3000 Belgium; 4 Leuven Brain Institute, KU Leuven, Leuven, 3000 Belgium

**Keywords:** Developmental biology, Genetics

## Abstract

Conditional protein tagging is a potent technique for elucidating protein expression patterns. The existing MiMIC and CRIMIC insertion collections in *Drosophila* genome offer a foundation for rapidly generating conditional protein tagging strains. In this study, we introduce dFlpTag, a new tool designed to create both constitutive and conditional protein tagging strains from MiMic or CRIMIC insertions. Moreover, dFlpTag enables co-labeling of the source cells of the protein of interest in a cell type-specific or sparse fashion. To demonstrate its utility, we generated strains for tagging the pre-synaptic protein Brp, the post-synaptic and polarity protein Dlg1, and the transmembrane protein Dpr12, thereby revealing their expression across multiple or a specific neuropil in the *Drosophila* central nervous system, individual synaptic boutons at the neuromuscular junction, and in large and small patches of epithelial cells within larval wing discs. We advocate for the straightforward application of this tool to tag thousands of genes that already possess MiMic or CRIMIC insertions within a coding intron.

## Introduction

Investigating a protein’s expression pattern is essential for a comprehensive understanding of its biological functions. In situ labeling offers a direct view of protein localization within tissues and cells, often relying on highly specific antibodies. When such antibodies are unavailable, a fusion protein with a standardized epitope tag can be used to proxy the protein’s distribution. The fusion protein could be encoded by a plasmid or a virus that is introduced into cells for transient expression, or by a transgene that is integrated in the genome for long-term expression. Additionally, the protein can be tagged with a fluorescent protein facilitating live imaging and enabling the study of fast dynamic changes in protein distribution, or with a tandem tag assisting protein purification and protein-protein interaction studies.

Another strategy of visualizing the expression pattern of a protein is to tag the endogenous protein, which is considered superior as it allows expression under the native regulatory elements. Tagging endogenous proteins requires genomic insertion of DNA sequences, which is technically challenging but has become increasingly feasible in *Drosophila* due to advancements in genetics. Early attempts to generate large-scale collections for screening endogenous protein tags in *Drosophila* involved mobilizing a transposal element carrying a protein-trap cassette^1^. Several groups have generated more than 500 protein-trap strains by using *P* or *PiggyBac* elements^2–4^. However, the position bias of these transposons obstructed further efforts to tag more genes^5^. Another approach is to integrate heterologous DNA into a precise genomic location using homology-directed repair (HDR). The adaptation of CRISPR-Cas9 in *Drosophila* has significantly improved the efficiency of creating precise double-strand DNA breaks and subsequent HDR^6–10^. This powerful tool was quickly adapted to generate endogenous protein tagged flies^11^. Despite the acceleration provided by CRISPR-Cas9, generating large-scale collections remains costly and labor-intensive, as specific gRNA and donor constructs are needed for each target gene.

The MiMIC (Minos-Mediated Integration Cassette) system offers an additional avenue for creating endogenous protein-trap lines. MiMIC is an engineered Minos transposon that can be swapped using recombinase-mediated cassette exchange (RMCE) for further gene editing^12^. Over 15,000 MiMIC strains have been created, each with a specific insertion, including 2854 MiMICs located in introns of 1862 genes that can be exchanged for a protein trap^13^. To expand the gene coverage, MiMIC-like cassettes, including CRIMIC (CRISPR Mediated Integration Cassette), were integrated into introns of specific genes through CRISPR-Cas9-directed insertion^11,14^. From these swappable insertions, more than 200 protein trap strains have been created for endogenous protein tagging^15^, with the potential for more in the future.

Constitutive protein tagging facilitates the visualization of a protein’s full distribution in all tissues. However, when a broadly expressed protein needs cell type-specific labeling to elucidate its biologic function or identify the cells that express the protein of interest, conditional protein tagging becomes essential. In the nervous system, synaptic proteins, including many secreted and cell surface proteins, are transported along axons and dendrites to regions far from the cell bodies. To reveal the details of their expression patterns, conditional protein tagging tools have been developed to tag axonal guidance and cell adhesion molecules^16^, neurotransmitter receptors^17–19^, synaptic vesicle makers^20,21^, and synapse scaffolding proteins^22^. Beyond personalized tools that requires individual construct design for each gene, the RMCE-based tool Flip-Flop and FlpTag has been developed for conditional protein tagging and could be easily applied to hundreds of genes by exchanging MiMIC and CRIMIC insertions^23,24^. These exiting genetic methods enable tagging of a protein of interest. However, additional tools, such as cell type-specific GAL4 drivers and UAS reporters, are needed to label and identify the source cells of the gene of interest. This becomes more challenging for a gene that is expressed in limited numbers of cells with its source cells unknown.

To address this problem, we have developed a new, generalizable method called dual FlpTag (dFlpTag) for simultaneously labeling endogenous proteins and visualizing source cells in *Drosophila*. We validated this method by generating constitutive and conditional tag versions for pre-synaptic protein Bruchpilot (Brp), post-synaptic and epithelial polarity protein Discs large 1 (Dlg1), and transmembrane protein Defective proboscis extension response 12 (Dpr12). Using constitutive dFlpTag tools, we demonstrated that GFP- or smGFP::HA tagged endogenous proteins matched previously published antibody staining and genetic labeling results. Conditional dFlpTag strains allowed cell-type specific or sparse labeling of endogenous proteins and their source cells by adapting different FLP expression strategies. Here we demonstrate the potential of dFlpTag to reveal the previously unknown expression profiles of many *Drosophila* genes with intronic MiMic or CRIMIC insertions.

## Results

### The design of the dual FlpTag system

**Fig. 1:**
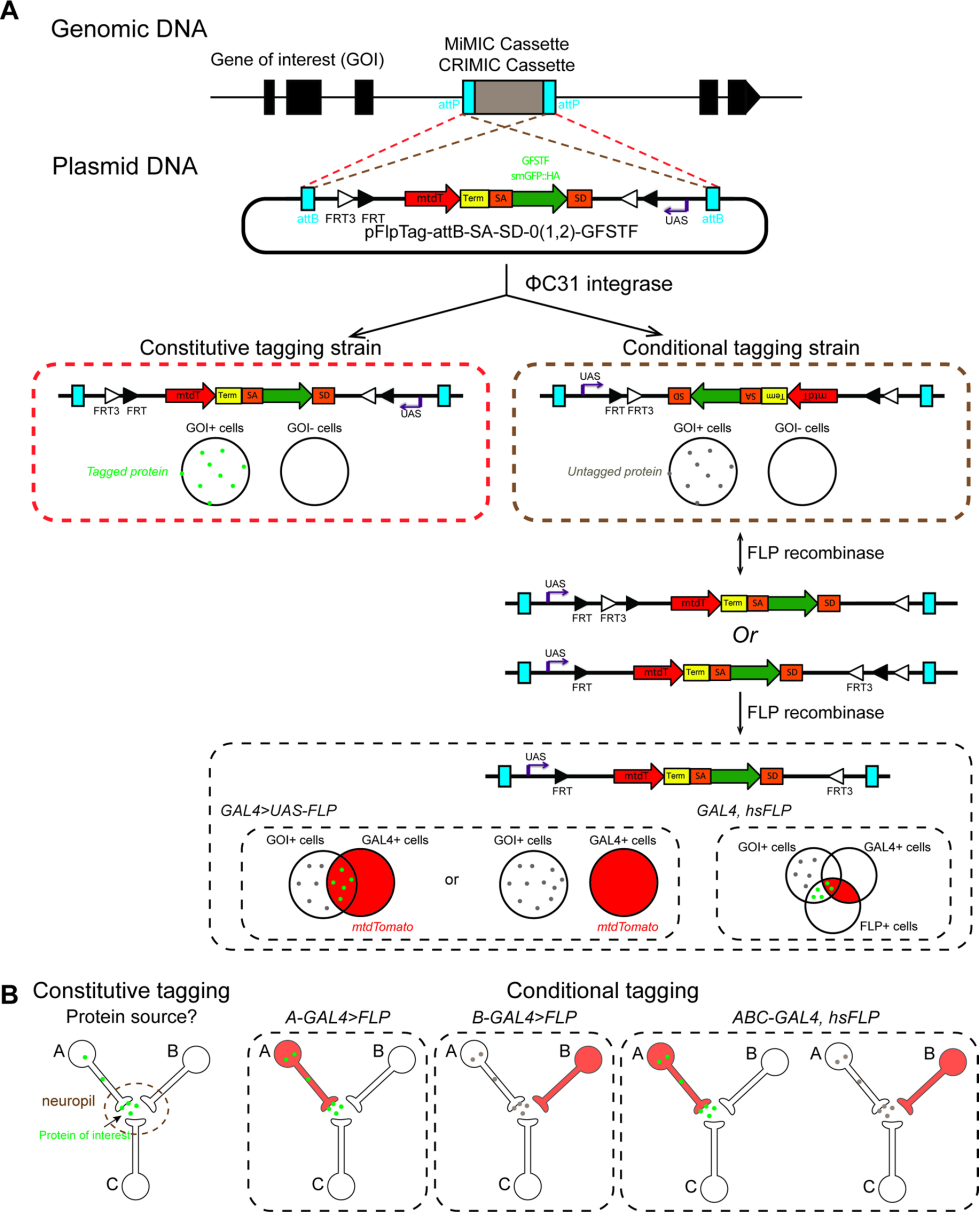
The dFlpTag system enables constitutive and conditional protein tagging and cell labeling. (**A**) The dFlpTag-attB constructs comprise attB sites, FRT3 and FRT sites, a protein trap (PT) module containing MHC splice acceptor (SA) and splice donor (SD) sites and a GFP reporter for protein tagging, as well as a 5xUAS and mtdTomato coding sequence for GAL4-driven cell labeling. The entire dFlpTag cassette can be integrated into a genomic locus of a gene of interest by swapping a MiMIC or CRIMIC cassette through recombinase-mediated cassette exchange (RMCE). The PT module allows for constitutive tagging of the protein of interest if the dFlpTag cassette aligns with gene transcription. If the dFlpTag cassette is integrated in a reversed orientation, the PT module can be reversed through FLP-mediated FRT and FRT3 recombination and is subsequently activated for conditional protein tagging. Meanwhile, mtdTomato ORF lies downstream of UAS promotor and can be expressed by a GAL4 driver for cell labeling. (**B**) Schematic illustration of dFlpTag application in the *Drosophila* central nervous system. A synaptic protein is transported to a specific neuropil, which is typically innervated by multiple neuronal types. By combining the conditional tagging strain with GAL4 drivers and either *UAS-FLP* or *hs-FLP*, both the target protein and its source cells can be labeled.

The dual FlpTag (dFlpTag) system is a novel strategy for protein tagging and cell labeling, enabling the simultaneous observation of the spatial distribution of an endogenous protein and the identification of the source cells of the labeled protein. This system incorporates a protein trap (PT) module, a red fluorescent protein mtdTomato (mtdT) coding sequence, two flanking inverted FRT and FRT3 recombination sites, and an upstream-activation sequence (UAS) (Fig. 1A). The PT module comprises a fluorescent protein tag, such as an EGFP-FLAsH-StrepII-3xFlag (GFSTF) tag or a signal-enhancing spaghetti monster GFP with multiple copies of the HA epitope tag (smGFP::HA), with splice acceptor (SA) and splice donor (SD) sequences at each end^1,2^. The PT module can be inverted as a whole through flippase-mediated recombination between FRT/FRT and FRT3/FRT3 sites. Upon inversion, mtdTomato is positioned downstream of the 5xUAS sequence and can be expressed in the presence of GAL4, a UAS-binding transcriptional factor.

For each protein tag (GFSTF or smGFP::HA), a set of three dFlpTag plasmid constructs are generated for each of the three possible open reading frames (0, 1, 2) (Fig. 1A). When a genomic Minos-Mediated Integration Cassette (MiMic) or CRISPR-Mediated Integration Cassette (CRIMIC) is present in an intron of a target gene, the dFlpTag plasmid for the corresponding reading frame can be selected and injected into the embryos of MiMIC/CRIMIC-bearing flies. The dFlpTag cassette integrates into the targeted intron through recombination between the injected plasmid and the MiMIC/CRIMIC. The integrated cassette can exist in two orientations relative to the transcription of the target gene. In the constitutive tagging orientation, the PT module aligns with target gene transcription, allowing expression of the endogenously GFP- or smGFP-tagged protein. The dFlpTag cassette can also be integrated in a conditional tagging orientation when the PT module is in the opposite orientation to the target gene transcription and does not affect the expression of the native endogenous protein. However, an endogenously GFP- or smGFP-tagged protein will be produced upon PT module inversion through FLP-mediated FRT/FRT3 recombination. FLP can be expressed in various ways; for example, hsFLP is expressed randomly by heat shock, and UAS-FLP is expressed in a tissue-specific manner when driven by specific GAL4 drivers. Thus, by employing different strategies to express FLP, the target protein can be tagged in various ways.

Beyond observing the expression of a target protein, identifying the source cells is often desirable, particularly in the nervous system where neurons have complex architectures and proteins can be transported far from the cell bodies (Fig. 1B). To facilitate this, a mtdTomato module is included for simultaneous cell labeling. Unlike the PT module, mtdTomato is not expressed in either constitutive or conditional strains until the entire dFlpTag cassette is inverted by FLP-mediated recombination. When GAL4 drivers are used to specifically express FLP in different candidate neuronal subtypes, the co-localization of mtdT and GFP-tag will help verify the source neurons. Alternatively, a more broadly expressed GAL4 driver could be used with hs-FLP to sparsely tag the target protein and label different cell types if cell type-specific GAL4 drivers are unavailable (Fig. 1B).

### dFlpTag labels pre-synaptic protein Brp

To ascertain the efficacy of the dFlpTag system in labeling endogenous proteins, we selected Bruchpilot (Brp), a pre-synaptic scaffolding protein commonly used for labeling *Drosophila* synapses and neuropils. Given the existence of tools for endogenous Brp tagging based on the MiMIC insertion *Brp*^*MI02987*^^15^, we injected dFlpTag-GFSTF or dFlpTag-smGFP::HA plasmids into *Brp*^*MI02987*^ embryos and successfully generated both constitutive and conditional strains for each construct.

**Fig. 2:**
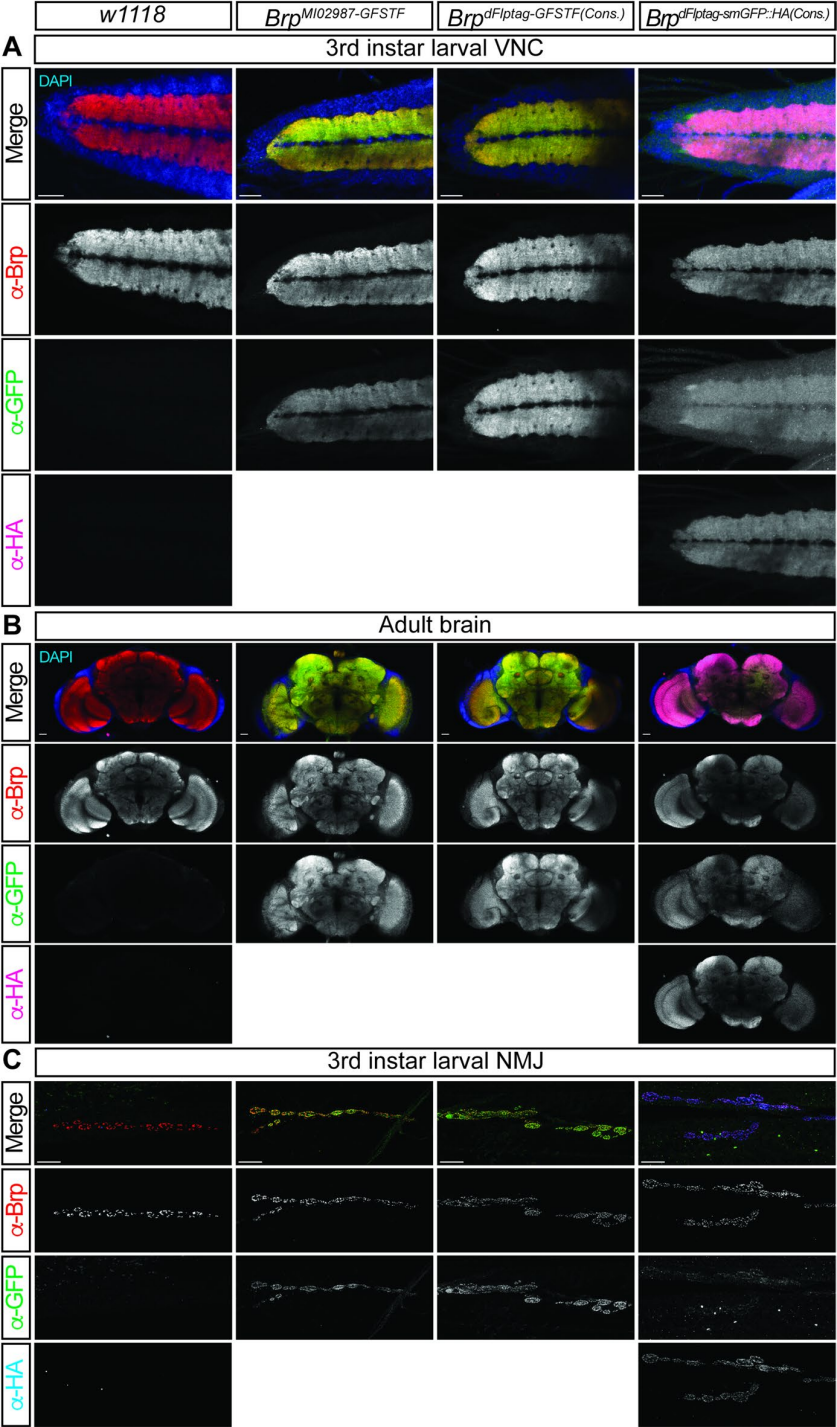
The dFlpTag system is used for constitutive Brp tagging for synaptic labeling. Constitutive GFSTF and smGFP::HA tagging strains were generated by swapping *Brp*^*MI02987*^. A&B) In larval ventral nerve cords (VNCs) (**A**) and adult brains (**B**), neuropils were specifically labeled by anti-Brp staining in all animals, or by anti-GFP staining in the previously published *Brp*^*MI02987−GFSTF*^ strain and newly-generated *Brp*^*dFlpTag−GFSTF(cons.)*^ and *Brp*^*dFlpTag−smGFP::HA(cons.)*^ strains. Anti-HA staining also labeled neuropils in the *Brp*^*dFlpTag−smGFP::HA(cons.)*^ strain with high specificity. (**C**) In 3rd instar larvae, pre-synaptic boutons of neuromuscular junctions (NMJ) were labeled by anti-Brp, anti-GFP and anti-HA staining in control and constitutive tagging strains. Scale bars: 20 μm in A, 50 μm in B, 10 μm in C.

We initially examined the global expression of GFSTF-tagged Brp in the larval and adult central nervous system of the constitutive strains (Fig. 2A and B). The GFSTF-tagged Brp was primarily detected in the neuropils of the larval ventral nerve cord (VNC) and adult brain, consistent with the previously published *Brp*^*MI02987-GFSTF*^ strain and anti-Brp (Nc82) staining. To further validate the expression of tagged Brp at the synaptic level, we assessed its expression pattern in the larval neuromuscular junction by staining the larval body wall muscles. The results showed that synaptic boutons and even individual active zones were made visible when labeled by the tagged Brp (Fig. 2C). For the smGFP::HA tag, the same GFP antibody gave weaker signals compared to the GFSTF tag. This is not surprising since the multiple copies of HA insertions with smGFP::HA changes GFP conformation, diminishes GFP fluorescence ability, and likely reduces the GFP antibody binding affinity. Meanwhile, the anti-HA staining yielded highly specific signals as expected (Fig. 2, A-C). Our data demonstrate that constitutive dFlpTag strains can label endogenous Brp with high specificity and accuracy.

**Fig. 3:**
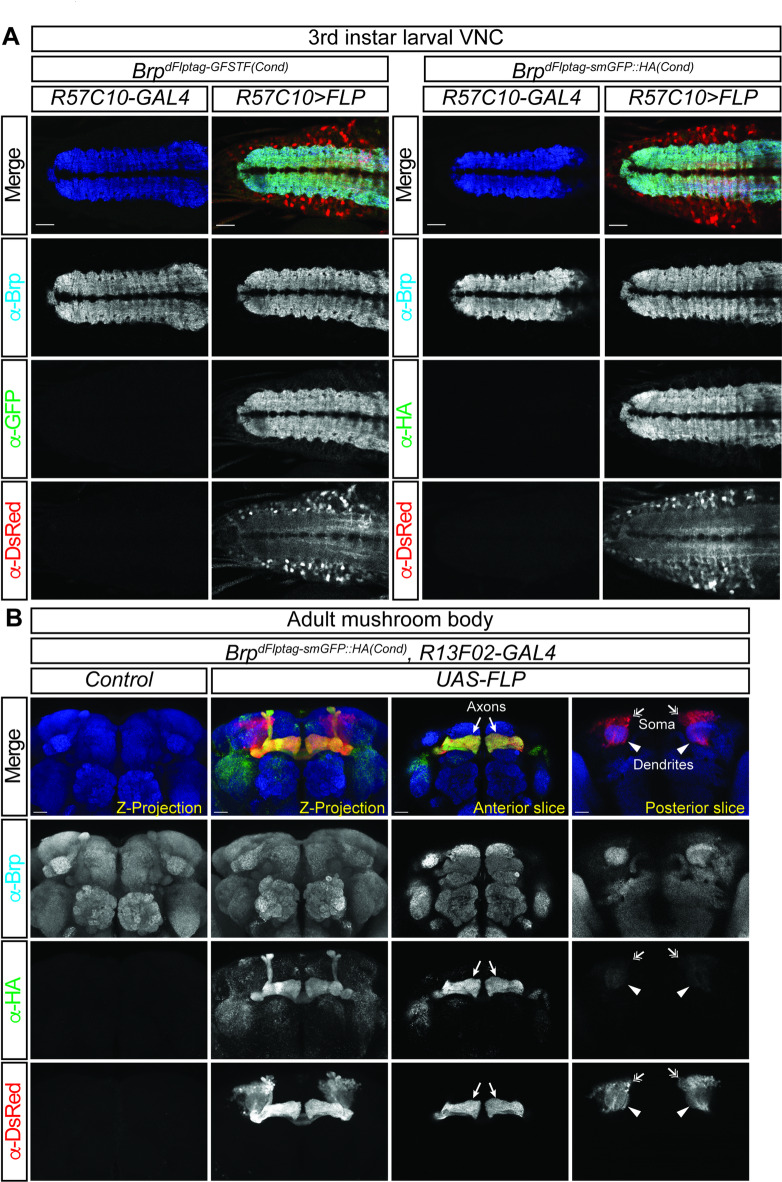
Conditional Brp tagging using the dFlpTag system. Conditional GFSTF and smGFP::HA tagging strains were generated by swapping *Brp*^*MI02987*^. (**A**) In larval VNCs, neuropils were specifically labeled by anti-GFP staining in the *Brp*^*dFlpTag−GFSTF(cond.)*^ strain, or by anti-HA staining in the *Brp*^*dFlpTag−smGFP::HA(cond.)*^ strain when *UAS-FLP* was expressed by pan-neuronal *R57C10-GAL4*. Neurons were also labeled by mtdTomato in these samples. (**B**) *R13F02-GAL4* was used to express *UAS-FLP* in the Kenyon cells (KCs) of the mushroom body (MB). In adult brains of *Brp*^*dFlpTag−smGFP::HA(cond.)*^ strain, FLP activated GAL4-driven mtdTamato expression for labeling the entire MB, while HA-tagged Brp was preferentially detected in KC axons (arrows) but not in KC dendrites (arrowheads) or soma (double arrows). Scale bars: 20 μm.

Subsequently, we conducted experiments to evaluate the conditional dFlpTag strains using the pan-neuronal driver *R57C10-GAL4*. In control animals lacking FLP expression, we detected no GFP or DsRed expression in the larval CNS (Fig. 3A), confirming that the conditional dFlpTag cassette cannot label endogenous proteins without FLP. Upon expression of *UAS-FLP* in neurons by *R57C10-GAL4*, GFP- or smGFP::HA-tagged Brp was detected in the neuropils of larval VNC (Fig. 3A). Concurrently, neuronal cell bodies and processes were labeled by DsRed. To illustrate neuronal subtype-specific labeling, we utilized the conditional smGFP::HA strain in conjunction with *R13F02-GAL4* to label Brp specifically in Kenyon cells (KCs) of the mushroom body (MB) (Fig. 3B). DsRed staining labeled KC soma (Fig. 3B, double arrows) and dendrites (Fig. 3B, arrowheads) on the posterior side of the adult brain, and the horizontal and vertical lobes of KC axons (Fig. 3B, arrows) on the anterior side of the brain, where they form synaptic connections with other mushroom body neurons. As anticipated, smGFP::HA-tagged Brp was predominantly observed in axons, similar to GFSTF-tagged Brp. These findings indicate that conditional dFlpTag strains can be employed to label endogenous pre-synaptic protein Brp in a cell-type-specific manner.

### dFlptag labels post-synaptic protein Disc-lethal giant 1 (Dlg1)

**Fig. 4:**
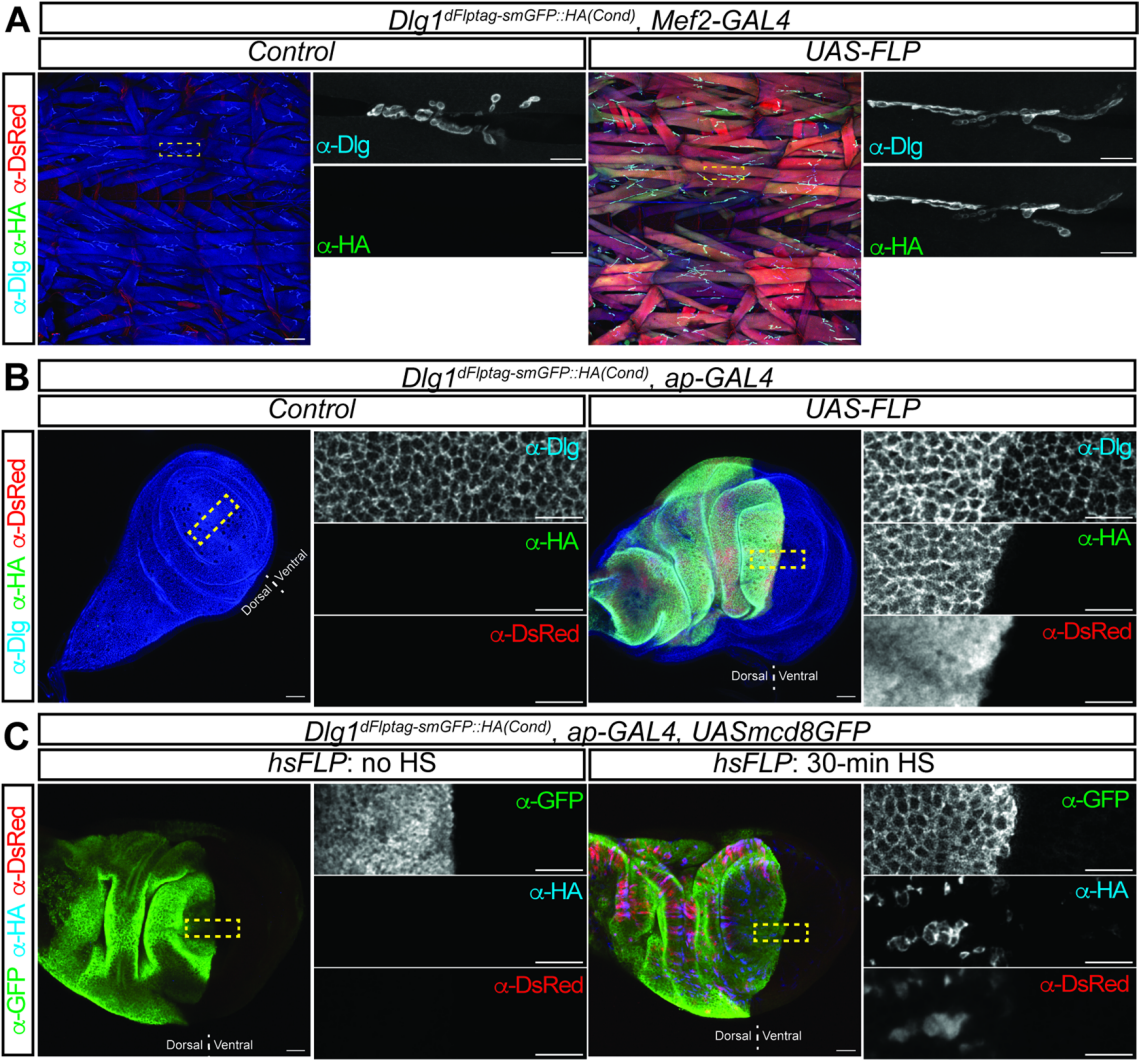
Conditional Dlg1 tagging using the dFlpTag system. A conditional smGFP::HA tagging strain was generated by swapping *Dlg1*^*MI06353*^. (**A**) In larval body wall muscles, anti-Dlg staining labeled the post-synaptic compartment of NMJs. By using *Dlg1*^*dFlpTag − smGFP::HA(cond.)*^ and *Mef2-GAL4 > FLP*, Dlg1 was also labeled by anti-HA staining in NMJs, and mtdTomato highlighted muscles. Scale bars: 100 μm in low magnification images and 20 μm in high magnification images. (**B**) In larval wing imaginal discs, Dlg1 is localized to epithelial cell membranes. With *ap-GAL4* driving *UAS-FLP*, Dlg1-smGFP::HA and mtdTomato were specifically expressed in dorsal epithelial cells of the wing pouch. (**C**) By using *hs-FLP*, Dlg1-smGFP::HA and mtdTomato expression were randomly activated and detected in patches of wing epithelial cells. Scale bars: 20 μm in low magnification images and 10 μm in high magnification images.

To further validate the effectiveness of the dFlpTag system in labeling post-synaptic proteins, we targeted *discs large 1* (*Dlg1*), which encodes a membrane-associated guanylate kinase (MAGUK) enriched in the postsynaptic compartment of the nervous system. Dlg1 is known to be crucial for clustering synaptic components and for synaptic development in neuromuscular junctions (NMJs)^25^. Employing a method similar to that used for *Brp*, a conditional *Dlg1* strain was generated by replacing *Dlg1*^*MI06353*^ with the dFlpTag-smGFP::HA cassette. To assess Dlg1 labeling in NMJs, immunostaining was performed on larval body wall muscles. In control animals without FLP expression, no signal was observed by anti-HA or anti-DsRed staining except that endogenous Dlg1 was detected by anti-Dlg1 staining in the muscles. When *Mef2-GAL4* was used to express FLP in muscles, anti-HA and anti-Dlg1 staining revealed highly overlapping expression patterns in the post-synaptic specializations of NMJs, with DsRed specifically detected in muscles (Fig. 4A).

Beyond the nervous system, Dlg1 functions as a tumor suppressor protein and regulates epithelial cell polarity by modulating the formation of septate junctions^26^. In larval wing imaginal discs, Dlg1 is located on cell membranes between epithelial cells, as clearly shown by anti-Dlg1 staining (Fig. 4B). Upon using *ap-GAL4* to express *UAS-FLP* in the dorsal half of the wing pouches, HA and DsRed staining was specifically detected in the corresponding regions of the wing disc, with the HA signal preferentially located in the cell membrane (Fig. 4B), suggesting that smGFP::HA tagged Dlg1 has correct subcellular localization in the epithelia. Interestingly, higher levels of Dlg1 were detected in the dorsal epithelia (dFlpTag ON side) compared to the ventral cells (dFlpTag OFF side), suggesting that the intronic insertion of the dFlpTag cassette could interfere with gene expression even in a opposite orientation related to host gene transcription.

In addition to *UAS-FLP*, we also explored random protein tagging using *hs-FLP* to activate the conditional dFlpTag cassette. After heat shocking the larvae, smGFP::HA tagged Dlg1 was observed in scattered epithelial cells in the wing discs (Fig. 4C). When *ap-GAL4* was used to express *UAS-mcd8GFP* and DsRed in the dFlpTag, GFP labeled the entire dorsal half of the wing pouch, but DsRed was restricted to the HA-positive dorsal epithelial cells (Fig. 4C). These results demonstrate that the dFlpTag system can be utilized to conditionally tag endogenous Dlg1 in various ways.

### dFlptag labels transmembrane protein Dpr12 in KC axon compartments

Building on the successful validation of the dFlpTag system for intracellular proteins Brp and Dlg1, we sought to extend our investigation to transmembrane proteins that are crucial for neural circuit formation. We chose Dpr12 due to its specific localization and function within the MB γ4 compartment. MB compartments are structured along KC axons to facilitate specific synaptic connections between KCs and distinct dopaminergic (DA) interneurons and MB output neurons within each compartment. The formation of these axonal compartments is regulated by unique pairs of transmembrane proteins, with Dpr12 (defective proboscis extension response 12) and its binding partner DIP-delta playing a key role in γ4/5 compartment development^27^.

**Fig. 5:**
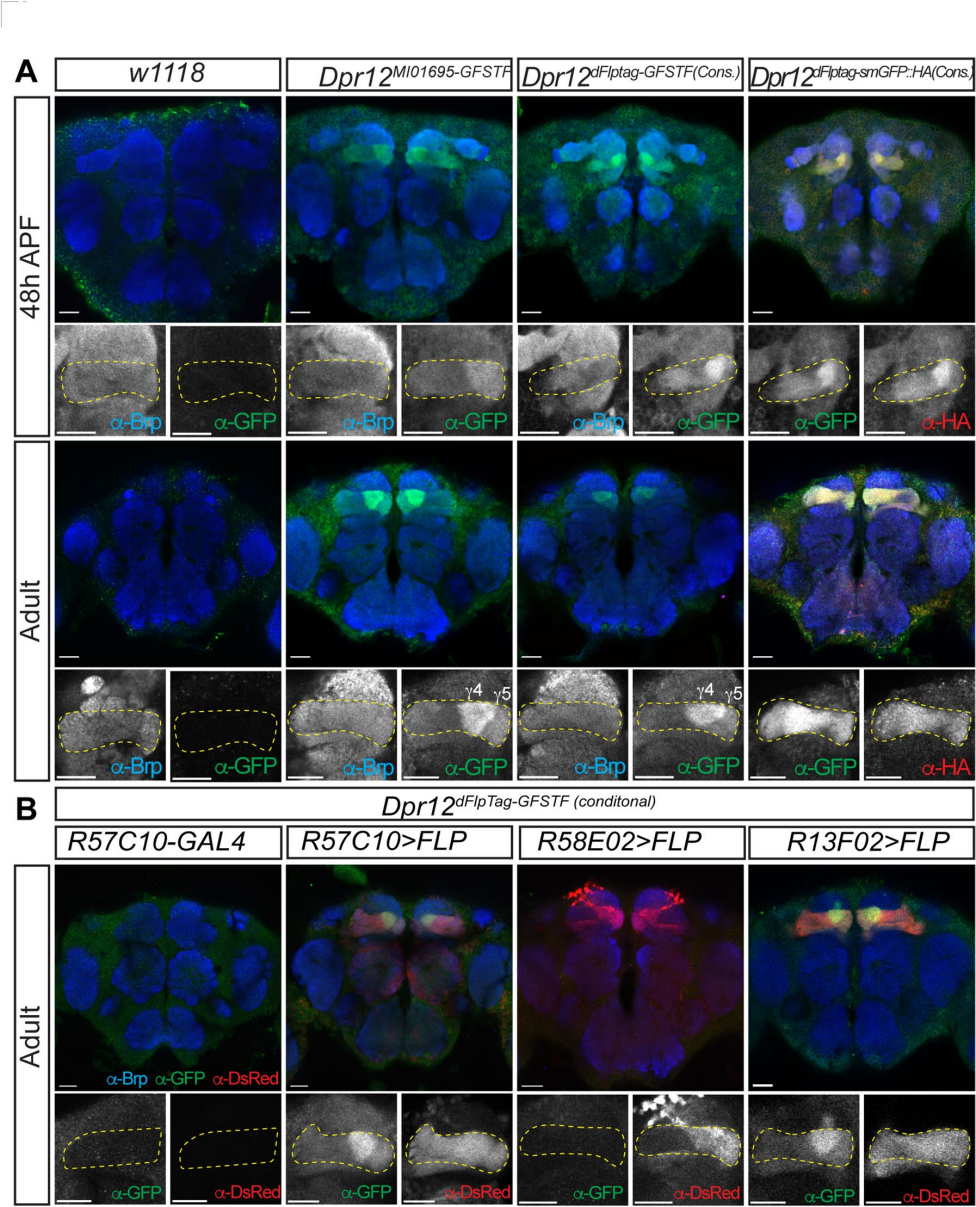
Tagging transmembrane protein Dpr12 in MB compartments using the dFlpTag system. Constitutive and conditional GFSTF and smGFP::HA tagging strains were generated by swapping *Dpr12*^*MI01695*^. (**A**) GFP-tagged Dpr12 was preferentially detected in MB γ4/γ5 compartments in both pupal and adult brains of constitutive tagging strains. (**B**) With different GAL4 drivers to express *UAS-FLP*, the corresponding cells were labeled by mtdTomato in *Dpr12*^*dFlpTag − GFSTF(cond.)*^ fly brains. However, the GFP-tagged Dpr12 was only detected with *R57C10-GAL4* and *R13F02-GAL4*, but not with *R58E02-GAL4*, which expresses in γ4/γ5-projecting dopamine neurons. Scale bars: 20 μm.

We successfully generated both constitutive and conditional dFlpTag strains for Dpr12. In the constitutive strains, GFSTF-tagged Dpr12 was enriched in the γ4/5 compartments of pupal and adult brains, mirroring the expression pattern observed in a previously published protein-trap strain (*Dpr12*^*MI01695-GFSTF*^) (Fig. 5A). The smGFP::HA tagged Dpr12 exhibited compartment-specific localization in pupal brains but surprisingly displayed a broader distribution in adult MB, suggesting that smGFP may somehow interfere with Dpr12 localization.

Given that each compartment is innervated by at least three types of neurons, identifying the cells expressing Dpr12 was essential for functional studies. To address this, we employed various GAL4 drivers to express FLP in specific MB neurons and activate the conditional dFlpTag cassette (Fig. 5B). When the pan-neuronal *R57C10-GAL4* or KC-specific *R13F02-GAL4* was used to express FLP, GFP-tagged Dpr12 was detected in the γ4 compartment. Conversely, no GFP signal was observed in the MB when *R58E02-GAL4* was used to express FLP in γ4/γ5 dopaminergic neurons. These results align with previous findings that Dpr12 is expressed by KCs but not by other MB projecting neurons^27^, and highlight the conditional dFlpTag as a potent tool for revealing the source cells of a protein of interest.

### Sparse labeling of Brp and Dlg1 in projection neurons

**Fig. 6:**
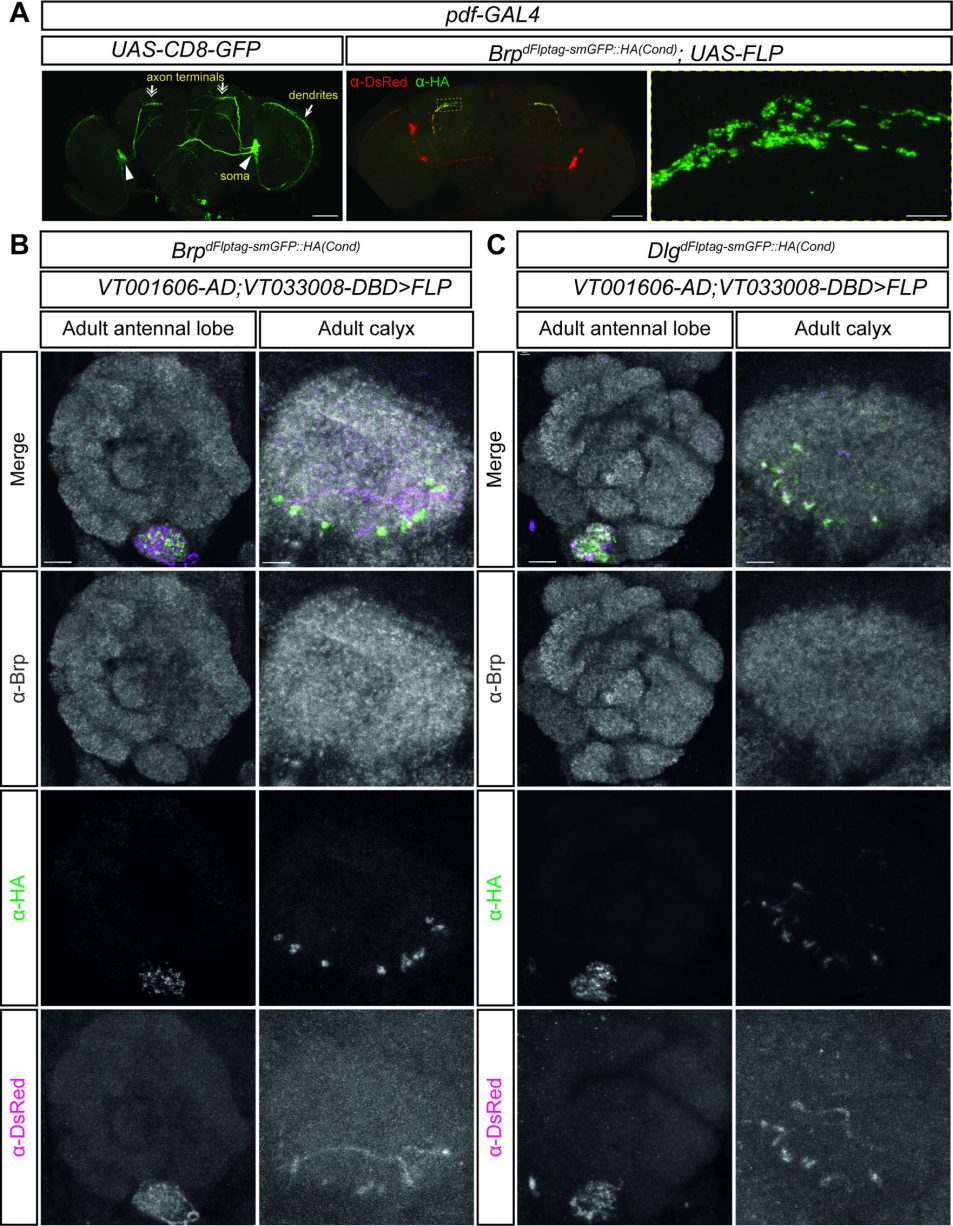
Sparse dFlpTag labeling in specific neuronal populations. (**A**) Expression of FLP under *pdf-GAL4* in four pairs of ventrolateral neurons drove HA-Brp labeling specifically in their axonal terminals. (**B**-**C**) A projection neuron-specific split-GAL4 driver (VT001606-AD; VT033008-DBD) was used to express FLP, labeling a single neuronal pair with mtdTomato. Their axonal and dendritic projections arborize in a single antennal lobe glomerulus and the mushroom body calyx. HA-tagged Brp (B) or Dlg1 (C) puncta were detected at these terminals. Scale bars: 50 μm (low-mag images in A) and 5 μm (high-mag images in A, B and C).

We validated the dFlpTag system for sparse neuronal labeling using two distinct GAL4 drivers. First, a pdf-GAL4 was used for targeting four large ventralateral neurons in each brain hemisphere (Fig. 6A). When *UAS-FLP* was expressed in these pdf neurons with conditional Brp^dFlpTag−smGFP::HA^, HA-tagged Brp puncta were specifically detected in the pre-synaptic terminals (Fig. 6A).

To target a sparser population that has a more sophisticated projection pattern, we used a projection neuron-specific split-GAL4 (VT001606-AD; VT033008-DBD). This driver labels a single pair of neurons with axonal and dendritic elaborations in the mushroom body calyx and one antennal lobe glomerulus, respectively. Expression of UAS-FLP resulted in specific neuronal labeling by DsRed. Meanwhile, HA-tagged Brp or Dlg1 punta were detected in the V glomerulus and calyx, precisely localizing to the DsRed-labeled axonal and dendritic structures (Fig. 6B and C). This result is consistant with the connectomic data that indicate these projection neurons have synaptic release sites and reciving synaptic inputs in both glomerulus and calyx. These results establish dFlpTag as a robust tool for precise, small-scale protein and neuronal labeling.

## Discussion

Here, we have developed the dual FlpTag (dFlpTag) system as a novel tool for both constitutive and conditional protein tagging. We have successfully generated constitutive and conditional tagging strains for the pre-synaptic protein Bruchpilot (Brp), the post-synaptic and epithelial polarity protein Discs large 1 (Dlg1), and the transmembrane protein Defective proboscis extension response 12 (Dpr12). For each of these proteins, the constitutive tagging strains exhibited expression patterns consistent with previously published antibody staining or constitutive tagging strains. Utilizing the conditional tagging strains, we have showcased cell-type specific protein tagging by employing multiple GAL4 drivers to express *UAS-FLP*, as well as sparse protein tagging using *hs-FLP*. These findings confirm the dFlpTag system’s potential as a new protein tagging tool, applicable to thousands of genes harboring MiMIC or CRIMIC insertions.

Unlike previous endogenous protein tagging tools, the dFlpTag cassette incorporates a mtdTomato coding sequence, which is activated by GAL4 only after FLP-mediated cassette inversion. When *UAS-FLP* is employed, mtdTomato is expressed in GAL4-positive cells, which may or may not express the target proteins. Consequently, the source cells can be identified through the expression of the tagged protein, when various GAL4 drivers targeting distinct cell types are used. This application has been demonstrated in the mushroom body for labeling Dpr12 with Kenyon cell or dopaminergic neuron-specific GAL4 drivers. Moreover, co-labeling of the target protein and source cells provides a direct visualization of the subcellular localization of the proteins, as evidenced by the conditional Brp labeling in KC axons and conditional Dpr12 labeling in specific KC axon compartments. However, it is important to note that GAL4 expression can be dynamic, and early GAL4 expression during development may not align with its expression pattern in later stages. In such cases, conditionally tagged proteins can be detected in cells that are GAL4 and mtdTomato negative at the time of observation, as observed with conditional Brp tagging using *R13F02-GAL4*.

FLP expression can also be activated through GAL4-independent methods. Here, we tested *hs-FLP*, which is expressed by a heat-shock promoter upon rapid temperature shifts. The heat shock induces stochastic cassette inversion and subsequent sparse protein tagging. The mtdTomato is also expressed in the intersectional cells for GAL4 expression and cassette reversion, as demonstrated by sparse Dlg1 tagging and epithelial labeling in wing discs. This sparse labeling offers at least two advantages. First, it can help elucidate the details of protein localization at the single-cell level. This could be particularly important for proteins mediating intercellular interactions. Second, when GAL4 drivers for specific candidate cell types are unavailable, a GAL4 driver with a broader expression spectrum can be utilized to generate stochastic co-labeling of a target protein and individual cells, which can help reveal the identity of the source cells.

Previous studies have shown that the internal insertion of GFSTF maintains normal protein function in 72% of cases^15^, suggesting that nearly 30% of GFSTF insertions could be dysfunctional. The effect of smGFP fusion has not been systematically examined. Although dFlpTag sucessfully labeled the three proteins and revealed their subcellular localization with both GFSTF or smGFP tags, smGFP-tagged Dpr12 loses its compartment-specific localization in adult brains, implying that smGFP may interfere with protein functions under certain conditions. The size difference between smGFP::HA (38.5 kDa) and GFSTF (33.5 kDa) could become significant for small proteins, like Dpr12 (37 kDa), in their localization and function. Since the subcellular localization of Dpr12 relies on the *in trans* interaction with DIP-delta, the change to the localization of smGFP-tagged Dpr12 suggests that smGFP may interfere with Dpr12/DIP-delta interactions. However, it remains unclear why smGFP-tagged Dpr12 is only mis-localized in adult but not in pupal brains. Other proteins, such as pupal specific adhesion molecules, could have a role in stabilizing Dpr12/DIP-delta binding or neuronal connections in the MB compartments. Therefore, GFSTF is safer for tagging a small protein and smGFP: HA is optimal for a protein with a low expression level.

While we have validated the application of dFlpTag in multiple experimental scenarios, it is important to note that the utility of our tool is tailored to the specific research objectives and experimental design. For studies where the primary goal is to comprehesively identify all source cells of a given protein across the entire organism or organ, employment of a ubiquitously and broadly expressed GAL4 driver is needed for conducting unbiased, whole-organism or whole-organ screening. For other studies focused on refining source cells to a targeted subset (e.g., a specific brain region, neuropil, or small group of candidate neuronal types), the selection of appropriate GAL4 drivers (ranging from those specific to the target cell population to more broadly acting ones, depending on the stage of the study) enables dFlpTag to efficiently and faithfully identify relevant source cells. However, it is critical to acknowledge that source cells lacking GAL4 expression may be underrepresented using this approach. To thoroughly elucidate the function of the targeted protein within particular cell types, supplementary genetic manipulations (e.g., gene mutation, RNAi, and transgene-mediated genetic rescue or overexpression) must be integrated with expression profiling analyses.

The application of dFlpTag is contingent upon the presence of a MiMIC, CRIMIC and other recombineering exchangeable cassettes within suitable coding introns. Bioinformatic analyses indicate that approximately 6000 genes in the *Drosophila* genome possess coding introns suitable for protein tagging^28^. Among them, near 2000 genes already possess a MiMIC insertion in their coding introns^15^. An ongoing project aims to expand this coverage by generating MiMIC-like cassette in thousands of additional genes using the CRISPR/Cas9 technique. This initiative is expected to significantly broaden the future application of the dFlpTag system.

## Methods and materials

### Fly strains and genetics

All Drosophila stocks and crosses were grown on cornmeal medium at 25 °C with a 12/12-hour light/dark cycle in specialized incubators (PGX-450 A, Saifu instruments) or climate controlled room (KU Leuven). The following strains and genetic tools were utilized in this study: *R57C10-GAL4* (BDSC#39171), *R13F02-GAL4* (BDSC#48571), *R58E02-GAL4* (BDSC#41347)^29,30^, *Mef2-GAL4* (BDSC#27390), *ap-GAL4* (BDSC#3041), *pdf-GAL4* (BDSC# 6899), *VT001606-AD* (BDSC# 72441), *VT033008-DBD*(BDSC# 71768), *UAS-FLP* (BDSC#4539), *hs-FLP*,* UAS-mCD8GFP* (BDSC#44408), *Brp*^*MI02987*^ (BDSC#37043), *Brp*^*MI02987−GFSTF*^ (BDSC#59292), *Dlg1*^*MI06353*^ (BDSC#41521), *Dpr12*^*MI01695*^ (BDSC#34237), *Dpr12*^*MI01695 − GFSTF*^ (BDSC#60171), *MCFO-1*^31^.

### Construction of pdFlpTag-attB-SA-SD-0(1,2)-GFSTF constructs

First, the pFlpStop-attB-UAS-2.1-tdTom (Addgene 88910)^32^ was digested with AgeI and PacI. The large fragment was recovered as the backbone vector, containing attB sites, FRT3 and FRT sites, mtdTomato coding sequence, a 5xUAS, hsp70 promotor and TATA box.

Next, the PT modules containing SA, SD and GFSTF coding sequence were PCR amplified from pBS-KS-attB1-2-PT-SA-SD-0(1,2)-EGFP-FIAsH-StrepII-TEV-3xFlag with custom primers (CCATAAATCTGAAAACCGGTAGTCGATCCAACATGGCGACTTG and CCTAGGATCGATTTAATTAAGTCGACAAGCTTAGAAGTTCAAATGGGC).

Last, the PCR products were cloned into the backbone vector using NEB high fidelity DNA assembly. Positive clones were verified by AgeI/PacI digestion and DNA sequencing.

### Construction of pdFlpTag-attB-SA-SD-0(1,2)-smGFP::HA constructs

The backbone vector was generated in the same way as the pdFlpTag-GFSTF constructs. The PT modules were composed of three fragments that were individually PCR amplified.

The SA sequence was amplified from pBS-KS-attB1-2-PT-SA-SD-0(1,2)-EGFP-FIAsH-StrepII-TEV-3xFlag with primers: CCATAAATCTGAAAACCGGTAGTCGATCCAACATGGCGACTTG and GGCACATCATAAGGGTAGGATCCGCCGCTACCTCC. The SD sequence was also amplified from pBS-KS-attB1-2-PT-SA-SD-0(1,2)-EGFP-FIAsH-StrepII-TEV-3xFlag with primers: CCTAGGATCGATTTAATTAA GTCGACAAGCTTAGAAGTTCAAATGGGC and ATGTCCCGGACTACGCTCGATCCGGAGGTAGCGGTG. The smGFP::HA coding sequence was amplified from the genomic DNA of MCFO-1 flies with the primers: TACCCTTATGATGTGCCCGATT and AGCGTAGTCCGGGACATCGTA. The three PT module fragments were simultaneously subcloned into the backbone using the NEB high fidelity DNA assembly. Positive clones were verified by AgeI/PacI digestion and DNA sequencing.

### Microinjection and screening of dFlpTag strains

The *dFlpTag* cassette was integrated into various MiMIC lines by RMCE, as previously described^12^. Briefly, *attB* plasmids carrying the *dFlpTag* cassette were injected into embryos derived from crosses between the desired MiMIC insertion line and flies expressing the phiC31 integrase^33^. Successful integration events were identified by the loss of the *yellow* marker. The orientation of the inserted *dFlpTag* cassette was confirmed by PCR using the following primers, as previously described^12^:

- **Orientation-MiL-F**: 5′-GCGTAAGCTACCTTAATCTCAAGAAGAG-3′.
- **FRTspacer_5p_rev**: 5′-AAATGGTGCAAAGAGAAGTTCC-3′.
- **FRTspacer_3p_for**: 5′-ACAATCCAGCTACCATTCTGC-3′.

For generating *dFlpTag* lines for *brp* and *dlg1*, embryo microinjections were performed in-house. The *dFlpTag* line for *dpr12* was generated by UniHuaii.com (Zhuhai, China).

### Immunohistochemistry and confocal imaging

*Drosophila* tissues were dissected in cold PBS and fixed with 4% PFA (Solarbio, P1112) for 20–30 min at room temperature. The samples were blocked in 5% Normal Donkey Serum in 0.3% PBST. After subsequent incubation with primary and secondary antibodies, samples were mounted in Fluoromount-G with DAPI (SouthernBiotech, 0100 − 20). Primary antibodies include chicken-anti-GFP (Abcam 13970), Rabbit-anti-DsRed (clontech 632496), Rat-anti-DsRed (Chromo Teck, 5f8), Mouse-anti-Brp (DSHB NC82), Mouse-anti-Dlg1 (DSHB 4F3), Rabbit-anti-HA (Cell signaling, C29F4),. Secondary antibodies include Alexa488, Alexa555, Alexa647 conjugated Donkey-anti-chicken (JacksonImmuno,703-545-155), Donkey-anti-Rabbit (Thermofisher, A32794), Donkey-anti-Mouse (Thermofisher, A32787), Donkey-anti-Rat (JacksonImmuno,712-605-153).

Immuno-stained samples were imaged using an inverted laser-scanning confocal microscope (Leica SP8) with a 20x air objective (HC PL APO 20x/0.75 CS2) or a 63x oil immersion objective lens (HC PL APO 63x/1.40 oil CS2).

### Heat shock experiment

For generating sparse labeling in larval wing imaginal discs, animals were reared at 25 °C. Second instar and early third instar larvae were collected and subjected to a brief heat shock in a 37 °C water bath for 30 min. Subsequently, animals were returned to a 25 °C incubator and raised for an additional two days before the wandering third instar were dissected.

## Data Availability

The sequences of the dFlptag plasmids generated during the current study are available in the GenBank (National Center for Biotechnology Information) repository, with accession numbers of PV098399 for pdFLPtag-SA-SD-0-GFSTF, PV099650 for pdFLPtag-SA-SD-1-GFSTF, PV099651 for pdFLPtag-SA-SD-2-GFSTF, PV099652 for pdFLPtag-SA-SD-0-smGFP-HA, PV099653 for pdFLPtag-SA-SD-1-smGFP-HA, PV099654 for pdFLPtag-SA-SD-2-smGFP-HA. Fly strains and plasmids are available upon request. Please contact X.X. (xiexj55@zju.edu.cn) and S.L. (sha.liu@kuleuven.be).
